# Supplementary material for: Socio-economic inequalities in burden of communicable and non-communicable diseases among older adults in India: Evidence from Longitudinal Ageing Study in India, 2017–18
Source: PLoS One. 2023 Mar 30;18(3):e0283385. doi: 10.1371/journal.pone.0283385 (PMC10062644; doi:10.1371/journal.pone.0283385)
Supplement: S1 Appendix — (DOCX) [file pone.0283385.s001.docx]

| **Risk factors of Communicable diseases** | **AOR (Adjusted Odds ratio)** | **95% confidence interval** |
| --- | --- | --- |
| **Age group** | | |
| 45-59® | 1 | [1,1] |
| 60-69 | 1.02 | [0.97,1.07] |
| >70 | 0.96 | [0.91,1.02] |
| **Sex** | | |
| Male® | 1 | [1,1] |
| Female | 1.04 | [0.99,1.09] |
| **Place of Residence** | | |
| Rural® | 1 | [1,1] |
| Urban | 0.86*** | [0.82,0.89] |
| **Marital Status** | | |
| Currently married® | 1 | [1,1] |
| Widowed | 0.97 | [0.83,1.14] |
| Others | 0.98 | [0.80,1.19] |
| **Education Level** | | |

###### Table S1. Logistic Regression Estimates for Older Adults who Suffered from Communicable Diseases by their Background Characteristics in India, 2017-18

| No Schooling® | 1 | [1,1] |
| --- | --- | --- |
| less than 5years | 0.98 | [0.92,1.05] |
| 5-9 years | 0.89*** | [0.84,0.94] |
| More than 10 years | 0.76*** | [0.71,0.81] |
| **Religion** | | |
| Hindu® | 1 | [1,1] |
| Muslim | 0.81*** | [0.76,0.87] |
| Christian | 1.13** | [1.03,1.23] |
| Others | 0.84*** | [0.78,0.88] |
| **Caste** | | |
| SC® | 1 | [1,1] |
| ST | 1.34*** | [1.25,1.45] |
| OBC | 1.20*** | [1.13,1.27] |
| Others | 0.94 | [0.88,1.00] |
| **Living Arrangements** | | |
| With spouse® | 1 | [1,1] |
| With spouse and Children | 1.085** | [1.02,1.15] |
| With children | 1.115 | [0.95,1.31] |
| Alone/others | 0.987 | [0.84,1.17] |
| **MPCE Quintile** | | |
| Poorest® | 1 | [1,1] |
| Poorer | 0.18*** | [0.11,0.26] |
| Middle | 0.18*** | [0.11,0.25] |
| Richer | 0.24*** | [0.16,0.32] |
| Richest | 0.31*** | [0.22,0.41] |
| **Body Mass Index** | | |
| Normal® | 1 | [1,1] |
| Underweight | 1.17*** | [1.11,1.23] |
| Over Weight | 0.91** | [0.87,0.96] |
| Obese | 0.95 | [0.91,1.00] |
| **Type of House** | | |
| Pucca® | 1 | [1,1] |
| Semi Pucca | 1.13*** | [1.07,1.19] |
| Kutcha | 1.14*** | [1.08,1.22] |
| **Source of Drinking Water** | | |
| Improved water® | 1 | [1,1] |
| Unimproved water | 1.25*** | [1.15,1.36] |
| **Type of Toilet Facility** | | |
| Improved toilet® | 1 | [1,1] |
| Unimproved | 1.21*** | [1.15,1.28] |
| **Cooking Fuel** | | |
| Clean Fuel® | 1 | [1,1] |
| Not Clean Fuel | 1.23*** | [1.18,1.29] |

| **Region** | | |
| --- | --- | --- |
| North® | 1 | [1,1] |
| Central | 1.24*** | [1.16,1.33] |
| East | 0.63*** | [0.59,0.67] |
| Northeast | 0.35*** | [0.32,0.39] |
| West | 0.54*** | [0.50,0.58] |
| South | 0.25*** | [0.23,0.27] |
| **Total** | **65,562** | |
| *Note: ® Reference category; 95% confidence intervals in brackets []; * p<0.05, ** p<0.01, *** p<0.001; SC: Scheduled Caste, ST: Scheduled Tribe, OBC: Other Backward Caste* | | |
