# Supplementary material for: Socio-economic inequalities in burden of communicable and non-communicable diseases among older adults in India: Evidence from Longitudinal Ageing Study in India, 2017–18
Source: PLoS One. 2023 Mar 30;18(3):e0283385. doi: 10.1371/journal.pone.0283385 (PMC10062644; doi:10.1371/journal.pone.0283385)
Supplement: S2 Appendix — (DOCX) [file pone.0283385.s002.docx]

| Table S2: Logistic Regression Estimates for Older Adults who Suffered from Non- Communicable Diseases by their Background Characteristics in India, 2017-18 | | |
| --- | --- | --- |
| **Risk Factors of Non-communicable Diseases** | **Adjusted odds ratio (AOR)** | **95% Confidence interval** |
| **Age group** | | |
| 45-59® | 1 | [1,1] |
| 60-69 | 1.57*** | [1.51,1.64] |
| >70 | 1.81*** | [1.71,1.92] |
| **Sex** | | |
| Male® | 1 | [1,1] |
| Female | 1.08*** | [1.03,1.14] |
| **Place of Residence** | | |
| Rural® | 1 | [1,1] |
| Urban | 1.42*** | [1.36,1.48] |
| **Education Level** | | |
| No Schooling® | 1 | [1,1] |
| less than 5 years | 1.28*** | [1.20,1.36] |
| 5-9 years | 1.24*** | [1.18,1.31] |
| More than 10 years | 1.33*** | [1.25,1.41] |
| **Religion** | | |
| Hindu® | 1 | [1,1] |
| Muslim | 1.36*** | [1.28,1.44] |
| Christian | 0.96 | [0.89,1.03] |
| Others | 1.13*** | [1.04,1.24] |
| **Caste** | | |
| SC® | 1 | [1,1] |
| ST | 0.65*** | [0.61,0.70] |
| OBC | 1.02 | [0.97,1.07] |
| Others | 0.96 | [0.91,1.02] |
| **Marital Status** | | |
| Currently married® | 1 | [1,1] |
| Widowed | 1.21** | [1.05,1.38] |
| Others | 1.00 | [0.84,1.20] |
| **Living Arrangements** | | |
| With spouse® | 1 | [1,1] |
| With spouse and children | 0.95 | [0.90,1.00] |
| With children | 0.88 | [0.77,1.02] |
| Alone/others | 0.81** | [0.70,0.94] |
| **MPCE Quintile** | | |
| Poorest® | 1 | [1,1] |
| Poorer | 1.20*** | [1.14,1.28] |
| Middle | 1.33*** | [1.25,1.41] |
| Richer | 1.52*** | [1.44,1.62] |
| Richest | 1.75*** | [1.65,1.86] |
| **Body Mass Index** | | |
| Normal® | 1 | [1,1] |

| Underweight | 0.67*** | [0.64,0.71] |
| --- | --- | --- |
| Overweight | 1.71*** | [1.64,1.80] |
| Obese | 2.22*** | [2.06,2.39] |
| **Physical Activities** | | |
| No® | 1 | [1,1] |
| Yes | 0.81*** | [0.78,0.84] |
| **Yoga** | | |
| No® | 1 |  |
| Frequently | 0.91 | [0.86,0.96] |
| **Mobility Restriction** | | |
| No restriction® | 1 | [1,1] |
| Mobility restriction | 2.06*** | [1.98,2.15] |
| **ADL Restriction** |  |  |
| No ADL restriction® | 1 | [1,1] |
| 1 or more ADL restriction | 1.41*** | [1.33,1.49] |
| **IADL Restriction** | | |
| No IADL restriction® | 1 | [1,1] |
| 1 or more IADL restriction | 1.22*** | [1.17,1.28] |
| **Tobacco Consumption** | | |
| No® | 1 | [1,1] |
| Smoke | 1.01 | [0.95,1.07] |
| Smokeless | 0.86 | [0.82,0.90] |
| Both | 0.94 | [0.84,1.04] |
| **Alcohol Consumption** | | |
| No® | 1 | [1,1] |
| Yes | 1.01 | [0.958,1.066] |
| **Region** | | |
| North® | 1 | [1,1] |
| Central | 0.64*** | [0.59,0.68] |
| East | 0.85*** | [0.80,0.91] |
| North-east | 0.71*** | [0.66,0.76] |
| West | 0.93* | [0.87,0.99] |
| South | 1.24*** | [1.17,1.31] |
| **Total** | **65,562** | |
| *Note: ® Reference category; 95% confidence intervals in brackets []; * p<0.05, ** p<0.01, *** p<0.001; SC: Scheduled Caste, ST: Scheduled Tribe, OBC: Other Backward Caste* | | |
