# Supplementary material for: Socio-economic inequalities in burden of communicable and non-communicable diseases among older adults in India: Evidence from Longitudinal Ageing Study in India, 2017–18
Source: PLoS One. 2023 Mar 30;18(3):e0283385. doi: 10.1371/journal.pone.0283385 (PMC10062644; doi:10.1371/journal.pone.0283385)
Supplement: S3 Appendix — (DOCX) [file pone.0283385.s003.docx]

**Table S3.** **Poor-Rich Ratio for communicable and non-communicable diseases among older adults across states in India, 2017–18**

| **State Name** | **Communicable Disease** | **Non-Communicable Disease** |
| --- | --- | --- |
|  | **Poor-Rich Ratio** | **Poor-Rich Ratio** |
| Jammu & Kashmir | 0.091 | 0.119 |
| Himachal Pradesh | 0.510 | 0.300 |
| Punjab | 0.225 | 0.200 |
| Chandigarh | 0.059 | 0.204 |
| Uttarakhand | 1.073 | 0.494 |
| Haryana | 0.941 | 0.500 |
| Delhi | 0.523 | 0.665 |
| Rajasthan | 1.483 | 0.577 |
| Uttar Pradesh | 2.739 | 0.849 |
| Bihar | 3.284 | 0.902 |
| Arunachal Pradesh | 0.530 | 0.390 |
| Nagaland | 0.253 | 0.258 |
| Manipur | 0.263 | 0.218 |
| Mizoram | 1.491 | 0.657 |
| Tripura | 0.935 | 0.284 |
| Meghalaya | 1.145 | 0.572 |
| Assam | 1.865 | 0.647 |
| West Bengal | 1.562 | 0.620 |
| Jharkhand | 3.066 | 0.750 |
| Odisha | 2.900 | 0.731 |
| Chhattisgarh | 6.346 | 1.503 |
| Madhya Pradesh | 1.280 | 0.580 |
| Gujarat | 0.932 | 0.525 |
| Daman & Diu | 0.478 | 0.375 |
| Dadra & Nagar Haveli | 2.461 | 0.694 |
| Maharashtra | 1.461 | 0.689 |
| Andhra Pradesh | 0.385 | 0.346 |
| Karnataka | 0.358 | 0.282 |
| Goa | 0.275 | 0.173 |
| Lakshadweep | 3.298 | 1.278 |
| Kerala | 0.565 | 0.493 |
| Tamil Nadu | 0.848 | 0.590 |
| Puducherry | 2.662 | 1.256 |
| Andaman & Nicobar | 0.935 | 0.302 |
| Telangana | 0.649 | 0.433 |
| **India** | **1.140** | **0.597** |
