# Supplementary material for: Socio-economic inequalities in burden of communicable and non-communicable diseases among older adults in India: Evidence from Longitudinal Ageing Study in India, 2017–18
Source: PLoS One. 2023 Mar 30;18(3):e0283385. doi: 10.1371/journal.pone.0283385 (PMC10062644; doi:10.1371/journal.pone.0283385)
Supplement: Attachment — Submitted filename: Point by point reply.docx [file pone.0283385.s005.docx]

Dear Editor,

Thank you for giving us the opportunity to revise the article. We have addressed all the raised pointed below.

**Editor’s comment:** We've checked your submission and before we can proceed, we need you to address the following issues:

1. We notice that your manuscript file was uploaded on December 14, 2022. Please can you upload the latest version of your revised manuscript as the main article file, ensuring that does not contain any tracked changes or highlighting.  This will be used in the production process if your manuscript is accepted. Please follow this link for more information: [http://blogs.PLOS.org/everyone/2011/05/10/how-to-submit-your-revised-manuscript/](http://blogs.plos.org/everyone/2011/05/10/how-to-submit-your-revised-manuscript/)
**Author’s Reply:** Thank you for pointing out the mistake. The updated manuscript has been uploaded in the portal.

**Editor’s comment:**  Please ensure that you refer to Figures 2 and 6 in your text as, if accepted, production will need this reference to link the reader to the figure.

**Author’s Reply:** We have thoroughly checked the manuscript and reference text for figure 2 was already present in the manuscript, however there is no figure 6 uploaded so there was nothing to mention about figure 6. We have only uploaded figure 1-5.

**Editor’s comment:** Please upload a copy of Figures 1 and 7, and 8 which you refer to in your text. Or if the figure is no longer to be included as part of the submission please remove all reference to it within the text.

**Author’s Reply:** Copy of figure 1 was already uploaded and checked in the pdf builder. There was no figure 7 and 8, neither we have mentioned that in our text, we have checked that thoroughly. The figures we have uploaded is restricted to figure 1- figure 5.
